# Mitral annular disjunction in Marfan syndrome: a multicenter cardiovascular magnetic resonance study

**DOI:** 10.1016/j.jocmr.2025.101938

**Published:** 2025-08-13

**Authors:** Kenan Kaya, Jonathan Kottlors, Thorsten W. Gietzen, Leon Bischoff, Jan M. Brendel, Reza Dehdab, Moritz C. Halfmann, Lukas Müller, Philipp v Stein, Lukas Goertz, Jan Paul Janßen, Roman Johannes Gertz, Robert Terzis, Vanessa Schmidt, Kilian Weiss, Christopher Hohmann, David Maintz, Tilman Emrich, Patrick Krumm, Julian A. Luetkens, Carsten H. Gietzen, Lenhard Pennig

**Affiliations:** a Institute for Diagnostic and Interventional Radiology, Faculty of Medicine and University Hospital Cologne, University of Cologne, Cologne, Germany; b Department III of Internal Medicine, Heart Center, Faculty of Medicine and University Hospital Cologne, University of Cologne, Cologne, Germany; c Department of Diagnostic and Interventional Radiology, University Hospital Bonn, Bonn, Germany; d Department of Radiology, Diagnostic and Interventional Radiology, University of Tübingen, Tübingen, Tübingen, Germany; e Department of Diagnostic and Interventional Radiology, University Medical Center of the Johannes Gutenberg-University, Mainz, Germany; f Philips GmbH Hamburg, Hamburg, Germany; g German Centre for Cardiovascular Research, Partner site Rhine-Main, Mainz, Germany; h Department of Radiology and Radiological Science, Medical University of South Carolina, Charleston, South Carolina, USA

**Keywords:** Marfan syndrome, Cardiovascular magnetic resonance, Mitral annular disjunction, Mitral valve prolapse, Connective tissue disorder

## Abstract

**Background:**

Data on the prevalence of mitral annular disjunction (MAD) in Marfan syndrome (MFS) based on cardiovascular magnetic resonance (CMR) is sparse. The purpose of this study was to assess prevalence, extent, and distribution of MAD in MFS using CMR and to examine its association with left heart parameters, aortic dimensions, and cardiovascular events.

**Methods:**

This retrospective multicenter study included CMR studies of patients treated for MFS at four tertiary care medical centers with a (likely) pathogenic fibrillin-1 gene variant. Two radiologists (5 and 8 years of experience in CMR) evaluated datasets for MAD (at 4 points around the annulus, including measurement of extent) and mitral valve prolapse (MVP). Further assessment comprised volumetric and functional analysis of the left ventricle (LV), left atrial size, and aortic root diameters. Cardiovascular events included aortic (aortic surgery or aortic dissection), arrhythmic (sustained ventricular tachycardia or sudden cardiac death), and mitral events (mitral valve surgery, MVS).

**Results:**

Among 91 patients [(28.9 ± 14.0 years, 47.3% female (n = 43/91)]81.3% (n = 74/91) had MAD (extent: 6.1 ± 2.6 mm). MAD was mostly found at the inferior insertion (72.5% of patients, n = 66/91) and usually affected all sites (39.6% of patients, n = 36/91). Left heart parameters and aortic dimensions did not differ between MAD and no MAD groups (P>0.05). MAD extent and localizations showed significant correlations with LV dilatation (e.g., inferior MAD: r = 0.62 for end-diastolic volume index), decreased LV ejection fraction (e.g., anterolateral MAD: r = −0.46), and MVP (e.g., MAD distance: r = 0.83), which was found in 44.6% of patients (n = 33/74) with MAD while only affecting 11.8% (n = 2/17) without MAD (P = 0.017). Based on receiver operating characteristic analysis for the prediction of MVP prevalence, a threshold of 7.1 mm MAD extent was identified as the optimal cut-off value (sensitivity: 77.1%, specificity: 89.3%). Additionally, subgroup analysis applying different thresholds of MAD extent revealed a significantly larger displacement of MVP and LV volumes as well as higher aortic root z scores for a threshold of ≥8 mm. After a mean follow-up of 4.0 ± 3.0 years, cardiovascular events (aortic: n = 13/91 [14.3%], arrhythmic: n =  2/91 [2.2%], and mitral: n = 2/91 [2.2%] of patients) did not differ significantly (all P>0.05) between no MAD and MAD groups regardless of applied thresholds although MVS was observed exclusively in patients with MAD.

**Conclusion:**

The high prevalence, large extent, and predominantly pan-annular distribution of MAD suggest a systemic annular pathology in MFS. Overall presence of MAD was not associated with changes to left heart parameters, aortic dimensions, and cardiovascular events. However, MAD, taking into account its extent and affected insertion sites, could serve as a potential marker of disease progression given the shown association of localizations and distance with LV dysfunction and remodeling as well as aortic enlargement and the formation of MVP.

## Graphical abstract

**Figure:**
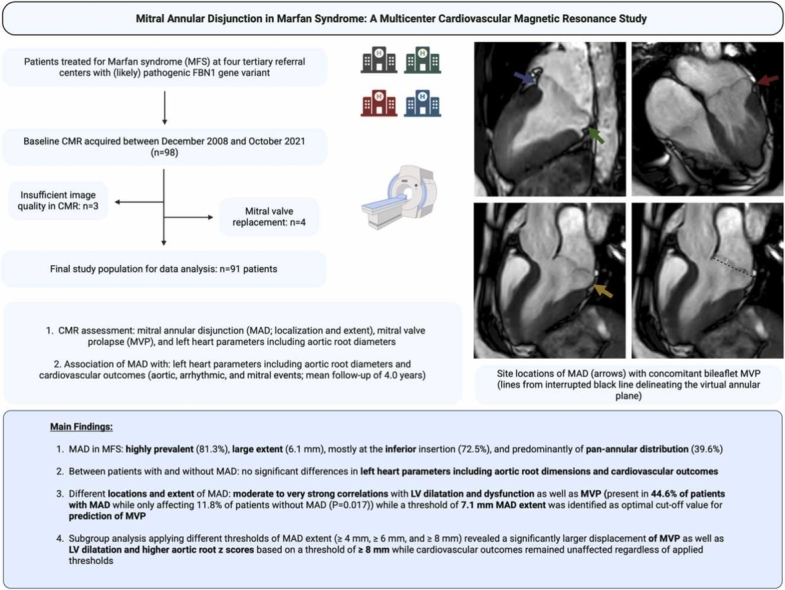
Created in BioRender. Kaya, K. (2025) https://BioRender.com/j6pwmjt.

## 1 Background

Marfan syndrome (MFS) is an inherited connective tissue disorder (CTD) with a reported prevalence of up to 17.2 per 100,000 individuals [1]. The majority of patients carries a (likely) pathogenic variant in the fibrillin-1 (FBN1) gene, which encodes for the FBN1 glycoprotein of the extracellular matrix [2]. As a multisystem disease, MFS primarily affects the cardiovascular, ocular, and skeletal organ systems [3]. Aortic root dilatation leading to aortic dissection and/or rupture [3] and mitral valve prolapse (MVP) represent the most common cardiovascular manifestations, with the latter occurring in 30%–60% of MFS patients [4]. Additionally, ventricular arrhythmia has been increasingly reported in MFS, presenting as non-sustained or sustained ventricular tachycardia (VT) and sudden cardiac death (SCD) [5], [6].

Regarding mitral valve abnormalities, mitral annular disjunction (MAD), referring to the distinct separation of the mitral valve annulus from the left ventricular (LV) myocardium, has recently gained substantial interest in research and clinical practice [7], [8], [9]. Based on the theory that the increased mobility of the annulus due to MAD leads to traction on the posterobasal LV myocardium and papillary muscles with subsequent injuries of the myocardium and fibrosis resulting in arrhythmic substrate [10], numerous studies suggest that MAD might cause arrhythmic events in patients with [11] or without MVP [12]. In MFS, MAD is highly prevalent and known to be associated with mitral valve surgery [8], [13], while a higher occurrence of arrhythmic events [8] and the necessity of aortic surgery at a younger patient age [13] are debated. However, previous studies investigating the prevalence of MAD in MFS almost exclusively used transthoracic echocardiography (TTE) for diagnosis mainly focusing on the inferolateral insertion [8], [13], [14] since this part of the annulus is best visualized in the parasternal long-axis view. Given the limited spatial resolution, discrete MADs may have been missed [15], [16], [17] and the involvement of other segments of the mitral valve annulus is unknown. In contrast, cardiovascular magnetic resonance (CMR) is not limited by body habitus while enabling a superior depiction of the annulus and delineation of MAD at all four insertion sites given its superior spatial resolution and soft tissue contrast [7], [12], [17].

The purpose of this study was to evaluate the prevalence, extent, and distribution pattern of MAD in MFS patients using multicenter CMR data and to examine its association with left heart parameters including aortic root dimensions and cardiovascular events.

## 2 Methods

### 2.1 Ethics

This retrospective multicenter study was part of the Radiological Cooperative Network project, which provides a collaborative environment for data exchange including all radiological departments of university hospitals in Germany. Given the retrospective study design and de-identification of patient data, this study was approved by the ethics committee of the leading center and the requirement for written informed consent was waived (24–1036-retro).

### 2.2 Study population

The authors retrospectively reviewed the internal databases at four tertiary care medical centers for patients treated for MFS based on the revised Ghent criteria [3]. Only patients with a (likely) pathogenic FBN1 gene variant who received annual clinical follow-up were eligible for this study. Patients younger than 16 years at first examination were defined as pediatric patients.

Participating centers are as follows: (1) Institute for Diagnostic and Interventional Radiology, Faculty of Medicine and University Hospital Cologne, University of Cologne, Cologne, Germany; (2) Department of Diagnostic and Interventional Radiology, University Hospital Bonn, Bonn, Germany; (3) Department of Radiology, Diagnostic and Interventional Radiology, University of Tübingen, Tübingen, Germany; (4) Department of Diagnostic and Interventional Radiology, University Medical Center of the Johannes Gutenberg-University, Mainz, Germany.

Patients were included if they underwent CMR comprising standard long- and short-axis cine sequences for assessment of the mitral valve and left ventricular function as well as magnetic resonance angiography (MRA) of the thoracic aorta between December 2008 and October 2021. The first available CMR was included for analysis. Examinations were excluded in case of (I) strong breathing artifacts in cine sequences or MRA leading to impaired assessment of the MV or the aortic root as determined by a board-certified cardiovascular radiologist with six years of experience in CMR (CG, level 2 equivalent of the European Association for Cardiovascular Imaging) and (II) mitral valve replacement.

The following data were retrieved from the medical charts from the first patient visit: Patient age, sex, cardiovascular risk factors, medication, aortic surgery, and aortic dissection prior to inclusion. Furthermore, patient age and medication were retrieved from the medical charts from the last patient visit.

### 2.3 CMR

All examinations were performed using commercially available whole body 1.5 and 3.0T MRI systems from different vendors. The imaging protocols comprised 2D balanced steady-state free precession cine sequences in standard orientations (4-chamber [4Ch], 2-chamber [2Ch], 3-chamber [3Ch], and short-axis) during end-expiratory breath-hold using retrospective electrocardiogram-gating prior to MRA. Cine sequence parameters are provided in Table S1 of the Supplementary Material. MRA was performed using either contrast-enhanced (3D CE-MRA, 4D CE-MRA, and steady-state 3D CE-MRA [18]) or non-contrast-enhanced (relaxation-enhanced angiography without contrast and triggering [19]) sequences as described elsewhere. Late gadolinium enhancement or T1 mapping sequences were not acquired.

### 2.4 Image analysis

Datasets were analyzed using a commercially available image viewer (DeepUnity Diagnost, release 1.1.1.1, Dedalus Healthcare Systems Group, Bonn, Germany) by a radiologist with 5 (K.K.) and a board-certified cardiovascular radiologist with 8 years of experience in CMR (LP, level 3 equivalent of the European Association for Cardiovascular Imaging) in consensus. Discrepancies were solved in consensus. Readers were blinded to clinical and outcome data.

#### 2.4.1 Mitral valve assessment

**Fig. 1:**
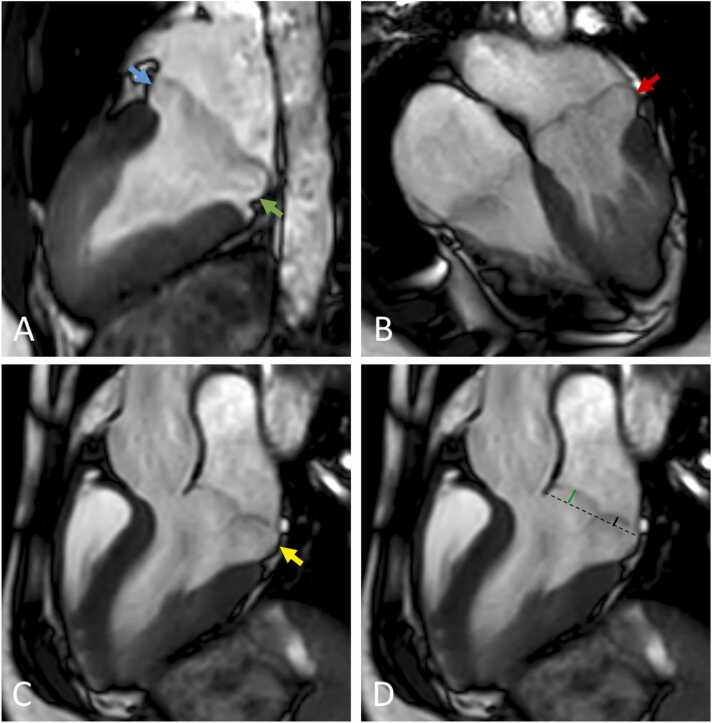
Cardiovascular magnetic resonance (2-chamber view (A), 4-chamber view (B), 3-chamber view (C and D) at end-systole) in a 29-year-old male with Marfan syndrome depicting mitral annular disjunction at all four sites (blue arrow: anterior, green arrow: inferior, red arrow: anterolateral, yellow arrow: inferolateral; A–C). Note the concomitant bileaflet mitral valve prolapse (black interrupted line: virtual annular plane, green line: anterior mitral leaflet, black line: posterior mitral leaflet; D)

For assessment of MAD, long-axis cine images (4Ch, 3Ch, and 2Ch) were analyzed for the presence of disjunction at the attachment of the posterior mitral leaflet to the anterior, anterolateral, inferolateral, and inferior segments of the annulus using standardized segmentation nomenclature (Fig. 1) [7], [20]. Following the consensus statement for CMR, MAD was defined as present when it measured ≥1 mm [21]. If MAD was observed, it was measured parallel to disjunction at end-systole from the top edge of the ventricular wall to the hinge of the leaflet from the left atrial wall [7]. Readers were specifically instructed to avoid false-positive findings (so-called “pseudo-MAD”, with the posterior mitral leaflet (PML) abutting the left atrial (LA) wall during systole) [16].

For assessment of prolapse or billowing of either leaflet of the mitral valve, the 3Ch view was used at end-systole as recommended by the American Society of Echocardiography. Billowing was classified as systolic protrusion of any part of the leaflet of <2 mm above the junctional plane. MVP was defined as systolic displacement of any part of the leaflet by ≥2 mm or more from the annular plane into the left atrium [22].

For assessment of the lengths of the anterior mitral leaflet and the PML, the 3Ch view was used at mid- or end-diastole. Assessing the last diastolic image where the mitral valve was clearly visible, leaflets were measured from their insertion to the most distal part [23].

#### 2.4.2 Left ventricular and left atrial assessment

Left ventricular volumes and ejection fraction were measured using the short-axis cine sequence. A commercially available software (IntelliSpace Portal version 10.1; Philips Healthcare, Best, the Netherlands) was used for post-processing analysis with automatic delineation of LV endo- and epicardial borders. LV end-diastolic (EDV), and LV end-systolic (ESV) volumes were divided by the body surface area (BSA) to obtain indexed values. The 4Ch view during LV end-systole was used for assessment of the left atrial (LA) area using planimetric measurement and divided by the BSA to obtain indexed values [24]. To calculate the BSA, the Mosteller formula was used [25].

#### 2.4.3 Aortic diameters

For assessment of aortic diameters in MRA, the manual Multiplanar-Reconstruction-tool in the image viewer was employed for measurement with manual perpendicular alignment to the vessel axis. Measurement of aortic diameters was performed at the annulus aortae, sinus of Valsalva, and sinotubular junction using an inner-edge approach. The z score for each MFS patient was calculated based on the diameter at the sinuses of Valsalva, age, and BSA. A z score between −2 and +2 was considered normal [26]. The equation was based on MRA-derived values as assessed by Kaiser et al [27].

### 2.5 Electrocardiography

Patients were scheduled to receive 12-lead electrocardiogram analysis at every patient visit at the treating tertiary care medical center. The 12-lead electrocardiogram closest to the most recent patient visit or prior to cardiac surgery was reviewed for analysis. Twenty-four-hour Holter monitoring was performed as clinically indicated based on the discretion of the treating cardiology consultant with the exam closest to the most recent patient visit or prior to cardiac surgery being used for further analysis.

### 2.6 Outcome

Outcome data were retrieved from the medical charts at the last available follow-up and included aortic events, any mitral valve surgery, arrhythmic events, and all-cause mortality. Aortic events comprised prophylactic and emergency aortic surgery (according to European Society of Cardiology guidelines on aortic disease) [28], and aortic dissection (Stanford A or B). Arrhythmic events were defined as sustained VT or SCD. Sustained VT was defined as a documented wide complex (QRS duration >120 ms) tachycardia (>100 beats per minute), for a duration of >30 s or accompanied by hemodynamic instability within 30 s [29]. SCD was defined as a witnessed cardiac arrest or death 1 h after symptom onset or unexpected death of a patient known to have been well within the last 24 h [29].

### 2.7 Statistical analysis

Statistical data analysis was performed using R version 3.6.2 on R studio version 1.2.5033.35 (https://cran.r-project.org/). A P-value <0.05 was considered statistically significant. Data are shown as absolute values with percentages or means with standard deviation, as appropriate. Variables were tested for normal distribution by the Shapiro-Wilk test. Patients with and without MAD were compared. Furthermore, a subgroup analysis for different MAD thresholds (≥4 mm, ≥6 mm, and ≥8 mm) was performed. For comparison of continuous variables, Student’s t-test or Mann-Whitney U-test, were employed, as appropriate. For comparison of non-parametric variables, χ²- or Fisher-test were used. Pearson´s correlation was used to measure the linear correlation between MAD insertion sites, MAD extent, and selected CMR parameters with a value of −1 meaning a total negative linear correlation, 0 being no correlation, and +1 referring to a total positive correlation. The interpretation of agreement was as follows: 0.00–0.19 very weak, 0.2–0.39 weak, 0.4–0.59 moderate, 0.6–0.79 strong, and 0.8–1.0 very strong agreement. To evaluate the diagnostic performance of MAD extent in predicting MVP, a receiver operating characteristic (ROC) analysis was performed.

## 3 Results

### 3.1 Study population and baseline characteristics

**Fig. 2:**
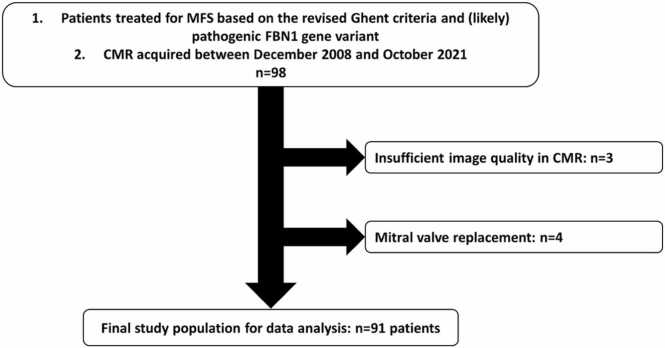
Workflow for inclusion and exclusion of patients. *CMR* cardiovascular magnetic resonance, *MFS* Marfan syndrome, *FBN1* fibrillin-1

Ninety-eight patients treated for MFS with CMR acquired between December 2008 and October 2021 were identified. After application of the exclusion criteria, the final study population consisted of 91 patients. The workflow for inclusion and exclusion of study participants is shown in Fig. 2. Eighteen pediatric patients were included in this study. Patients were almost exclusively examined using 1.5T systems (98.9%, n = 90/91) while the thoracic aorta was predominantly depicted using CE-MRA techniques (65.9%, n = 60/91). Further details are provided in Table S2 of the Supplementary Material.

Among 91 patients (28.9 ± 14.0 years, 43 (47.3%, [n = 43/91]female), 74 (81.3%,[n= 74/91]) had MAD. Patient characteristics did not differ between patients with and without MAD (all P>0.05).

Detailed information regarding patient characteristics is given in Table 1.

**Table 1:** Characteristics of all patients, patients with MAD, and patients without MAD.

Patient characteristics	Overall (n = 91)	Patients with MAD (n = 74)	Patients without MAD (n = 17)	P value
Age at baseline, mean±SD [years]	29.0 ± 14.0	28.6 ± 14.1	30.9 ± 16.1	0.654
Pediatric patients, n (%)	18/91 (19.8)	13/74 (17.6)	5/17 (29.4)	0.443
Female, n (%)	43/91 (47.3)	35/74 (47.3)	8/17 (47.1)	1.0
BSA at CMR, mean±SD [m^2^]	1.9 ± 0.4	1.9 ± 0.4	2.0 ± 0.4	0.568
*Cardiovascular risk factors at baseline, n (%)*				
Hypertension	20/91 (22.0)	16/74 (21.6)	4/17 (23.5)	0.738
Diabetes mellitus	4/91 (4.4)	4/74 (5.4)	0/17 (0)	1.0
Smoking	10/91 (11.0)	9/74 (12.2)	1/17 (5.9)	1.0
Dyslipidemia	5/91 (5.5)	5/74 (6.8)	0/17 (0)	0.583
Family history of Marfan syndrome	24/91 (26.4)	20/74 (27.0)	4/17 (23.5)	1.0
*Medication at baseline, n (%)*				
Beta-blocker	37/91 (40.7)	31/74 (41.9)	6/17 (35.3)	1.0
Angiotensin receptor blocker	21/91 (23.1)	17/74 (23.0)	4/17 (23.5)	0.750
ACE inhibitor	10/91 (11.0)	9/74 (12.2)	1/17 (5.9)	1.0
Diuretics	5/91 (5.5)	4/74 (5.4)	1/17 (5.9)	1.0
Calcium channel blockers	1/91 (1.1)	1/74 (1.4)	0/17 (0)	1.0
Anticoagulants	12/91 (13.2)	10/74 (13.5)	2/17 (11.8)	1.0
Age at last follow-up, mean±SD [years]	33.4 ± 15.7	32.7 ± 14.9	36.8 ± 15.7	0.337
Follow-up duration, mean±SD [years]	4.0 ± 3.0	4.2 ± 3.0	3.3 ± 2.9	0.372
*Medication at last follow-up, n (%)*				
Beta-blocker	44/91 (48.4)	37/74 (50.0)	7/17 (41.2)	1.0
Angiotensin receptor blocker	30/91 (33.0)	24/74 (32.4)	6/17 (35.3)	0.817
ACE inhibitor	12/91 (13.2)	8/74 (10.8)	4/17 (23.5)	0.118
Diuretics	14/91 (15.4)	11/74 (14.9)	3/17 (17.6)	0.700
Calcium channel blockers	5/91 (5.5)	4/74 (5.4)	1/17 (5.9)	1.0
Anticoagulants	19/91 (20.9)	15/74 (20.3)	5/17 (29.4)	0.461
Received 12-lead electrocardiography, n (%)	85/91 (93.4)	70/74 (94.6)	15/17 (88.2)	1.0
Received at least one 24-hour Holter monitoring, n (%)	13/91 (14.3)	10/74 (13.5)	3/17 (17.6)	0.690
Prophylactic aortic surgery prior to inclusion, n (%)	12/91 (13.2)	8/74 (10.8)	4/17 (23.5)	0.118
Emergency aortic surgery prior to inclusion, n (%)	5/91 (5.5)	4/74 (5.4)	1/17 (5.9)	1.0
Stanford type A dissection prior to inclusion, n (%)	6/91 (6.6)	5/74 (6.8)	1/17 (5.9)	1.0
Stanford type B dissection prior to inclusion, n (%)	3/91 (3.3)	2/74 (2.7)	1/17 (5.9)	0.433

*ACE* angiotensin-converting enzyme, *BSA* body surface area, *CMR* cardiovascular magnetic resonance, *SD* standard deviation, *MAD* mitral annular disjunction.

Data are presented as mean ± standard deviation for continuous variables and as number (percentage) for categorical variables, unless otherwise specified.

### 3.2 CMR characteristics of patients with and without MAD

MVP was present in more than a third of patients (38.5%, n = 35/91), yielded a balanced distribution between leaflets, and affected 44.6% (n = 33/74) of patients with MAD while it only occurred in 11.8% (n = 2/17) of patients without MAD (P = 0.017). Billowing of the mitral valve was only found in patients with MAD (24.3%[n = 18/74] of patients) and affected both leaflets to the same extent. Mitral valve length, LV parameters, LA size, and aortic diameters did not differ between patients with and without MAD.

Detailed results regarding CMR characteristics of patients with and without MAD are given in Table 2.

**Table 2:** CMR characteristics of all patients, patients with MAD, and patients without MAD.

CMR parameter	Overall (n = 91)	Patients with MAD (n = 74)	Patients without MAD (n = 17)	P value
*Mitral valve*				
Prolapse, any leaflet, present, n (%)	35/91 (38.5)	33/74 (44.6)	2/17 (11.8)	**0.013**
Anterior leaflet, present, n (%)	27/91 (29.7)	25/74 (33.8)	2/17 (11.8)	
mean±SD [mm], if present	4.2 ± 2.0	4.3 ± 2.1	3.5 ± 1.1	0.486
Posterior leaflet, present, n (%)	25/91 (27.5)	24/74 (32.4)	1/17 (5.9)	
mean±SD [mm], if present	4.0 ± 1.3	3.9 ± 1.3	5.2	0.445
Bileaflet, present, n (%)	14/91 (15.4)	13/74 (17.6)	1/17 (5.9)	0.179
Billowing, any leaflet, present, n (%)	18/91 (19.8)	18/74 (24.3)	0/17 (0.0)	**0.020**
Anterior leaflet, present, n (%)	11/91 (12.1)	11/74 (14.9)	0/17 (0.0)	
mean±SD [mm], if present	1.4 ± 0.3	1.4 ± 0.3	0/17 (0.0)	n/a
Posterior leaflet, present, n (%)	11/91 (12.1)	11/74 (14.9)	0/17 (0.0)	
mean±SD [mm], if present	1.6 ± 0.2	1.6 ± 0.2	0/17 (0.0)	n/a
Bileaflet, present, n (%)	4/91 (4.4)	4/74 (5.4)	0/17 (0.0)	0.190
*Mitral valve length*				
Anterior mitral leaflet, mean±SD [mm]	25.1 ± 5.9	25.2 ± 5.8	24.6 ± 6.2	0.725
Posterior mitral leaflet, mean±SD [mm]	15.2 ± 4.3	15.2 ± 4.5	15.0 ± 3.8	0.943
*Left ventricular parameters*				
Ejection fraction, mean±SD [%]	61.4 ± 9.8	62.2 ± 8.0	57.7 ± 14.7	0.185
End-diastolic volume index, mean±SD [ml/m^2^]	80.2 ± 36.8	77.6 ± 26.1	91.4 ± 64.3	0.541
End-systolic volume index, mean±SD [ml/m^2^]	33.7 ± 31.7	30.8 ± 15.4	46.1 ± 64.5	0.485
Mass index, mean±SD [g/m^2^]	52.8 ± 17.6	52.7 ± 17.7	53.1 ± 16.6	0.819
*Left atrium*				
Area/BSA, mean±SD [cm^2^/m^2^]	11.2 ± 3.0	11.2 ± 3.1	11.4 ± 2.7	0.931
*Aortic diameters*				
Aortic annulus, mean±SD [mm]	30.9 ± 5.1	30.6 ± 4.6	32.0 ± 6.8	0.619
Sinus of Valsalva, mean±SD [mm]	39.8 ± 7.3	39.6 ± 7.2	40.6 ± 7.9	0.589
Aortic root z score, mean±SD	5.3 ± 3.1	5.3 ± 3.1	5.5 ± 3.1	0.763
Aortic root z score >2, n (%)	77/91 (84.6)	62/74 (83.8)	15/17 (88.2)	0.500
Sinotubular junction, mean±SD [mm]	31.4 ± 6.6	30.9 ± 5.8	33.5 ± 9.0	0.324

*CMR* cardiovascular magnetic resonance, *SD* standard deviation, *MAD* mitral annular disjunction.

Data are presented as mean ± standard deviation for continuous variables and as number (percentage) for categorical variables, unless otherwise specified.

Bold indicates statistical significance.

### 3.3 Prevalence, extent, and distribution pattern of MAD

Overall, 364 insertion sites of the annulus were analyzed, of which MAD affected 231 localizations (63.5%, n = 231/364), yielding an extent of 6.2 ± 2.6 mm. MAD was most commonly located at the inferior insertion point (72.5% of patients, n = 66/91) while the remaining sites showed a comparable prevalence. MAD findings measuring >6 mm were mostly found at the inferior insertion (47,2 %, n = 43/91).

**Fig. 3:**
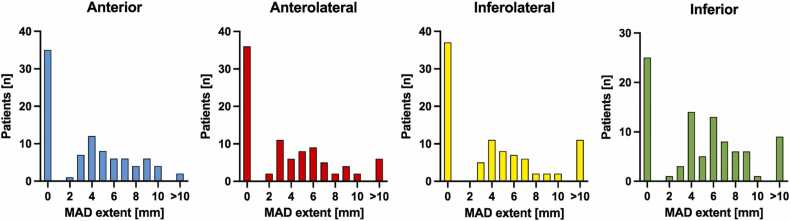
Histograms displaying the distribution of MAD extent for the four insertion points. *MAD* mitral annular disjunction

Detailed information regarding the prevalence and extent of MAD is given in Table 3. Histograms displaying the distribution of MAD extent for the four insertion points are provided in Fig. 3.

**Table 3:** Prevalence and extent of MAD in all analyzed 91 patients shown separately for each site and for all sites combined given for any extent and for selected thresholds.

MAD anterior, present, n (%)	56/91 (61.5)
mean±SD [mm], if present	6.0 ± 2.5
≥ 4 mm, n (%)	51/91 (56.0)
≥ 6 mm, n (%)	39/91 (42.8)
≥ 8 mm, n (%)	27/91 (29.6)
MAD anterolateral, present, n (%)	55/91 (60.4)
mean±SD [mm], if present	6.1 ± 3.1
≥ 4 mm, n (%)	49/91 (53.8)
≥ 6 mm, n (%)	37/91 (40.6)
≥ 8 mm, n (%)	26/91 (28.5)
MAD inferolateral, present, n (%)	54/91 (59.3)
mean±SD [mm], if present	7.0 ± 3.6
≥ 4 mm, n (%)	53/91 (58.2)
≥ 6 mm, n (%)	42/91 (46.1)
≥ 8 mm, n (%)	28/91 (30.7)
MAD inferior, present, n (%)	66/91 (72.5)
mean±SD [mm], if present	6.9 ± 3.2
≥ 4 mm, n (%)	61/91 (67.0)
≥ 6 mm, n (%)	43/91 (47.2)
≥ 8 mm, n (%)	28/91 (30.7)
MAD all findings, n (%)	231/231 (100.0)
mean±SD [mm]	6.2 ± 2.6
≥ 4 mm, n (%)	214/231 (92.6)
≥ 6 mm, n (%)	161/231 (69.6)
≥ 8 mm, n (%)	109/231 (47.1)

*SD* standard deviation, *MAD* mitral annular disjunction.

Data are presented as mean ± standard deviation for continuous variables and as number (percentage) for categorical variables, unless otherwise specified.

If present, MAD primarily affected all insertion sites (39.6% of all patients, n = 36/91) while manifestations at one localization rarely occurred (10.9% of all patients, n = 10/91).

Detailed results regarding the distribution patterns of MAD are given in Table 4.

**Table 4:** Distribution patterns of MAD.

Number of sites of observed MAD: n (%)	Distribution of MAD when detected	Patients, n (%)
0: 17 (18.7)	No site affected	17/91 (18.7)
1: 10 (10.9)	Anterior	2/91 (2.2)
	Anterolateral	5/91 (5.5)
	Inferolateral	1/91 (1.1)
	Inferior	2/91 (2.2)
2: 11 (12.1)	Anterior and anterolateral	1/91 (1.1)
	Anterior and inferolateral	0/91 (0)
	Anterior and inferior	7/91 (7.7)
	Anterolateral and inferolateral	1/91 (1.1)
	Anterolateral and inferior	2/91 (2.2)
	Inferolateral and inferior	0/91 (0)
3: 18 (19.8)	Anterior, anterolateral, inferolateral	1/91 (1.1)
	Anterior, anterolateral, inferior	2/91 (2.2)
	Anterior, inferior, inferolateral	6/91 (6.6)
	Anterolateral, inferolateral, inferior	8/91 (8.8)
4: 36 (39.6)	All sites	36/91 (39.6)

*MAD* mitral annular disjunction.

Data are presented as number (percentage).

### 3.4 Correlation between the localization and extent of MAD with CMR parameters

Structural and functional parameters of the left heart yielded significant correlations with the different localizations of MAD and their extent. Specifically, increased LV EDV index was strongly associated with inferior MAD (r = 0.62) and yielded a moderate association with the extent of MAD (r = 0.57). Increased LV ESV index showed moderate associations with all insertion sites (r = 0.48–0.53), except for anterior MAD (r = 0.34), and extent of MAD (r = 0.53). Decreased LVEF was predominantly observed in anterolateral (r = −0.46) and inferolateral (r = −0.39) MAD. Increased LA size yielded weak associations with inferior (r = 0.35) and anterior (r = 0.32) MAD.

MVP yielded a very strong correlation with MAD extent (r = 0.83) and was mostly observed in inferolateral (r = 0.78), anterior (r = 0.69), and inferior (r = 0.65) MAD while there was only a weak correlation between anterolateral MAD and MVP (r = 0.34).

Correlation between aortic dimensions and localization and extent of MAD were predominantly non-significant with only enlarged aortic annulus (inferior MAD: r = 0.27) and increased z score (extent: r = 0.30, anterior MAD: r = 0.28) showing significant, but weak correlations.

**Fig. 4:**
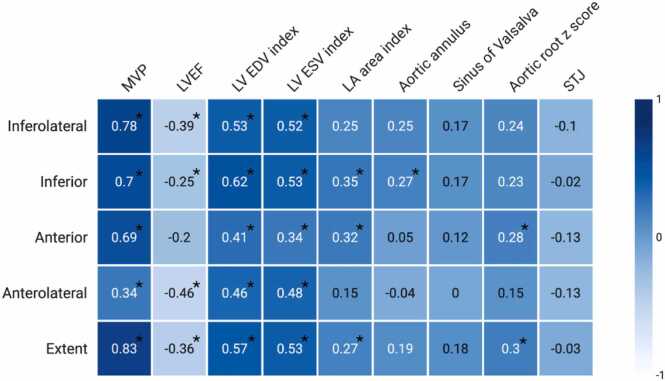
Pearson correlation between the localization and extent of mitral annular disjunction with selected cardiovascular magnetic resonance parameters. Asterisk indicates statistical significance. *BSA* body surface area, *EDV* end-diastolic volume, *ESV* end-systolic volume, *LA* left atrium, *LV* left ventricle, *LVEF* left ventricular ejection fraction, *MVP* mitral valve prolapse, *STJ* sinotubular junction

Fig. 4 depicts the correlation between localization and extent of MAD with selected CMR parameters.

### 3.5 Outcome

**Fig. 5:**
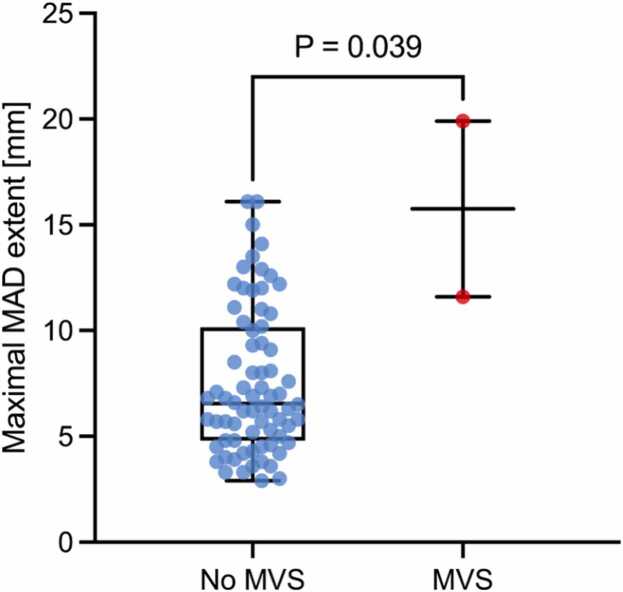
Maximal MAD extent in patients with MAD who did (n = 2) and did not (n = 72) undergo MVS. Box represents the IQR, the horizontal lines indicate the median, and whiskers extend to the minimum and maximum values. All individual data points are overlaid as scattered dots. *MAD* mitral annular disjunction, *MVS* mitral valve surgery, *IQR* interquartile range

Overall, 13 aortic events occurred during follow-up (14.3% [n = 13/91]of patients), which did not differ significantly between patients harboring MAD (n = 11/91, 14.9% of patients with MAD) and those without MAD (n = 2/91, 11.8% of patients without MAD; P>0.05). One aortic dissection occurred in the MAD group, classified as Stanford B. Despite occurring exclusively in patients with MAD, performed mitral valve surgery and all-cause mortality did not yield statistically significant difference between both groups (all P>0.05). Patients in which MVS was observed during follow-up yielded bileaflet MVP and pan-annular MAD with extents of up to 11.6 and 19.9 mm (both at the inferior insertion), respectively. Consequently, patients treated with MVS showed a significantly larger MAD extent than those patients harboring an MAD, who did not receive surgery (P = 0.0039, Fig. 5). Sustained arrhythmic events did not differ between groups (P>0.05). The only observed arrhythmic event in the MAD group occurred in a patient harboring an isolated inferior MAD with 5.7 mm distance.

Detailed results are presented in Table 5.

**Table 5:** Outcome of patients for all patients, patients with MAD, and patients without MAD.

	Overall (n = 91)	Patients with MAD (n = 74)	Patients without MAD (n = 17)	P value
Aortic events, n (%)	13/91 (14.3)	11/74 (14.9)	2/17 (11.8)	0.888
Prophylactic aortic surgery, n (%)	12/91 (13.2)	10/74 (13.5)	2/17 (11.8)	1.0
Aortic dissection, n (%)	1/91 (1.1)	1/74 (1.4)	0/17 (0)	1.0
Mitral valve surgery, n (%)	2/91 (2.2)	2/74 (2.7)	0/17 (0)	1.0
Sustained VT or SCD, n (%)	2/91 (2.2)	1/74 (1.4)	1/17 (5.9)	1.0
All-cause mortality, n (%)	2/91 (2.2)	2/74 (2.7)	0/17 (0)	1.0

*VT ventricular tachycardia, SCD sudden cardiac death, MAD mitral annular disjunction.*

Data are presented as number (percentage).

### 3.6 Subgroup analysis for MAD extent

For further analysis, subgroups were established based on the extent of MAD (≥4 mm, ≥6 mm, and ≥8 mm thresholds). Detailed results regarding the prevalence of MAD overall and for different insertion sites based on these thresholds are given in Table 3. Of note, the different extents of MAD based on these thresholds were equally balanced between the insertion sites. Following above referenced thresholds for the largest MAD per patient, selected CMR parameters and outcome data were compared between patients harboring MAD of ≥4 mm, ≥6 mm, and ≥8 mm extent, respectively, and remaining patients (with lesser MAD extent and without MAD).

**Fig. 6:**
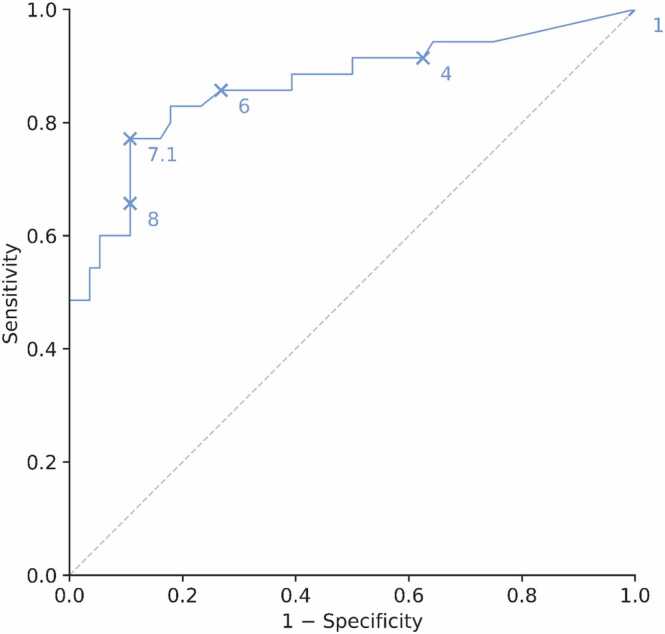
ROC curve evaluating the discriminatory performance of maximal MAD extent (in mm) for the presence of mitral valve prolapse. The analysis yielded an AUC of 0.87. The optimal cut-off, determined by the Youden index, was 7.1 mm, corresponding to a sensitivity of 77.1% and a specificity of 89.3%. Thresholds of 1 mm, 4 mm, 6 mm, and 8 mm are additionally indicated. *ROC* receiver operating characteristic, *AUC* area under the curve

Regarding the comparison of CMR parameters between the different groups, Table 6 gives detailed results. Patients with MAD ≥4 mm, ≥6 mm, and ≥8 mm all showed a significantly higher prevalence of MVP and bileaflet MVP compared to remaining patients. Furthermore, MAD ≥8 mm yielded a significantly larger displacement of the AML and PML in case of MVP. Based on ROC analysis for the prediction of MVP prevalence, a threshold of 7.1 mm MAD extent was identified as the optimal cut-off value for predicting MVP, yielding a sensitivity of 77.1% and a specificity of 89.3% (Fig. 6). Regarding LV remodeling, end-diastolic volume index increased with MAD extent and reached statistical significance for the ≥6 mm and ≥8 mm thresholds. Likewise, end-systolic volume index yielded significantly higher values at the ≥8 mm threshold. Patients with MAD ≥8 mm yielded lower LVEF and larger LA size although without statistical significance. However, patients with MAD ≥8 mm harbored a significantly larger aortic root z-score.

**Table 6:** Comparison of selected CMR parameters for patients yielding MAD with ≥4 mm, ≥6 mm, and ≥8 mm extent compared to remaining patients (with lesser MAD extent and without MAD).

CMR parameters	MAD ≥ 4 mm (n = 66)	Other (n = 25)	P value	MAD ≥ 6 mm (n = 45)	Other (n = 46)	P value	MAD ≥ 8 mm (n = 29)	Other (n = 62)	P value
*Mitral valve*									
Prolapse, any leaflet, present, n (%)	32/66 (48.5)	3/25 (12.0)	**0.003**	30/45 (66.7)	5/46 (10.9)	**<0.001**	23/29 (79.3)	12/62 (19.4)	**<0.001**
Anterior leaflet, present, n (%)	25/66 (37.9)	2/25 (8.0)		23/45 (51.1)	4/46 (8.7)		20/29 (69.0)	7/62 (11.3)	
mean±SD [mm], if present	4.3 ± 2.1	3.5 ± 1.5	0.487	4.3 ± 2.2	3.5 ± 0.9	0.561	4.6 ± 2.2	3.1 ± 0.8	**0.017**
Posterior leaflet, present, n (%)	23/66 (34.8)	2/25 (8.0)		22/45 (48.9)	3/46 (6.5)		17/29 (58.6)	8/62 (12.9)	
mean±SD [mm], if present	4.1 ± 1.4	3.7 ± 2.1	0.726	4.1 ± 1.3	3.2 ± 1.7	0.258	4.4 ± 1.2	3.2 ± 1.5	**0.038**
Bileaflet, present, n (%)	16/66 (24.2)	1/25 (4.0)	**0.034**	15/45 (33.3)	2/46 (4.3)	**<0.001**	14/29 (48.3)	3/62 (4.8)	**<0.001**
*Left ventricular parameters*									
Ejection fraction, mean±SD [%]	61.5 ± 7.1	61.2 ± 14.9	0.578	61.1 ± 7.6	61.6 ± 11.6	0.448	59.7 ± 6.6	62.1 ± 10.9	0.121
EDVI, mean±SD [ml/m^2^]	79.8 ± 26.4	81.2 ± 56.9	0.278	84.2 ± 28.2	76.2 ± 43.9	**0.018**	93.2 ± 28.4	74.1 ± 39.1	**<0.001**
ESVI, mean±SD [ml/m^2^]	32.1 ± 15.1	37.8 ± 56.3	0.202	34.0 ± 16.3	33.4 ± 42.1	0.084	38.7 ± 16.6	31.4 ± 36.8	**0.001**
*Left atrium*									
Area/BSA, mean±SD [cm^2^/m^2^]	11.3 ± 3.1	10.9 ± 3.0	0.603	11.7 ± 3.2	10.8 ± 2.3	0.150	12.0 ± 3.6	10.8 ± 2.6	0.123
*Aortic diameters*									
Aortic annulus, mean±SD [mm]	31.1 ± 4.7	30.2 ± 6.5	0.253	30.9 ± 4.9	30.9 ± 5.5	0.937	31.9 ± 4.9	30.4 ± 5.3	0.168
Sinus of Valsalva, mean±SD [mm]	40.1 ± 6.8	38.9 ± 8.8	0.549	40.0 ± 7.2	39.5 ± 7.6	0.775	41.7 ± 6.8	38.8 ± 7.5	0.206
Aortic root z score, mean±SD	5.5 ± 2.9	4.8 ± 3.5	0.356	5.7 ± 2.9	5.0 ± 3.2	0.302	6.5 ± 2.6	4.7 ± 3.1	**0.006**
Aortic root z score >2, n (%)	57/66 (86.4)	20/25 (83.3)	0.740	38/45 (84.4)	39/46 (86.7)	1.0	28/29 (96.6)	49/62 (80.3)	0.054
Sinotubular junction, mean±SD [mm]	31.1 ± 5.9	32.2 ± 8.5	0.556	31.9 ± 7.2	30.9 ± 6.1	0.401	30.9 ± 6.5	31.6 ± 6.7	0.323

*CMR* cardiovascular magnetic resonance, *EDVI* end-diastolic volume index, *ESVI* end-systolic volume index, *SD* standard deviation, *MAD* mitral annular disjunction.

Data are presented as mean ± standard deviation for continuous variables and as number (percentage) for categorical variables, unless otherwise specified.

Bold indicates statistical significance.

Regarding patient outcome, no significant differences were found between the groups. Detailed results are given in Table 7.

**Table 7:** Outcome of aortic for patients yielding MADs with ≥4 mm, ≥6 mm, and ≥8 mm extent compared to remaining patients (with lesser MAD extent and without MAD).

	MAD ≥ 4 mm (n = 66)	Other (n = 25)	P value	MAD ≥6 mm (n = 45)	Other (n = 46)	P value	MAD ≥8 mm (n = 29)	Other (n = 62)	P value
Aortic events, n (%)	9/66 (13.6)	4/25 (16.0)	0.730	6/45 (13.3)	7/46 (15.9)	0.788	6/29 (20.6)	7/62 (11.3)	0.511
Prophylactic aortic surgery, n (%)	8/66 (12.1)	4/25 (16.0)	0.504	5/45 (11.1)	7/46 (15.9)	0.756	5/29 (17.2)	7/62 (11.3)	0.509
Aortic dissection, n (%)	1/66 (1.5)	0/25 (0)	1.0	1/45 (2.2)	0/46 (0)	1.0	1/29 (3.4)	0/62 (0)	0.318
Mitral valve surgery, n (%)	2/66 (3.1)	0/25 (0)	1.0	2/45 (4.4)	0/46 (0)	0.493	2/29 (6.9)	0/62 (0)	0.099
Sustained VT or SCD, n (%)	1/66 (1.5)	1/25 (4.0)	0.448	0/45 (0)	2/46 (4.3)	0.494	0/29 (0)	2/62 (3.2)	1.0
All-cause mortality, n (%)	1/66 (1.5)	1/25 (4.0)	0.457	0/45 (0)	2/46 (4.3)	0.494	0/29 (0)	2/62 (3.2)	1.0

*CMR* cardiovascular magnetic resonance, SD standard deviation.

Data are presented as number (percentage).

## 4 Discussion

In this multicenter study, the prevalence and distribution pattern of MAD in MFS patients was investigated using CMR data and correlated with changes to left heart parameters including aortic dimensions and the occurrence of cardiovascular events. MAD was highly prevalent, mostly located at the inferior insertion, and predominantly of pan-annular distribution. Left heart parameters and aortic dimensions did not differ between patients with and without MAD. However, localization and extent of MAD yielded distinct correlations with LV dilatation and dysfunction as well as the presence of MVP, which almost exclusively occurred in MAD with an extent of 7.1 mm serving as an optimal cut-off value for the prediction of MVP. Accordingly, patients with MADs ≥8 mm yielded significantly larger displacement of MVP, increased LV volumes, and higher aortic root z scores. Regarding patient outcome, cardiovascular events did not differ between patients with or without MAD regardless of applied thresholds for MAD extent.

While patient management in CTD mainly focuses on timing of aortic surgery and prevention of aortic dissection, mitral valve involvement including MVP and MAD is gaining increased interest as a potential source of morbidity [30]. To our knowledge, the present study is the first to assess the prevalence of MAD in MFS at all four insertion sites using multicenter CMR data in a predominantly adult patient cohort. Doan et al. also included CMR data in a retrospective study to investigate the prevalence of MAD in MFS [14]. However, this single-center study exclusively included pediatric patients with diagnosis of MFS not requiring genetic testing, while CMR, being used for confirmation of initial diagnosis of MAD in TTE, was only performed in a small subset of patients (40%) and limited to the evaluation of inferolateral MAD [14]. Additionally, the study mainly focused on the progression of MAD during childhood and did not investigate clinical outcome [14].

In the present work, MAD was found in 81% (n = 74/91) of genetically confirmed MFS patients with an extent of 6 mm. In contrast, studies using TTE reported a lower prevalence of 34%–60% with a distance of 2–8 mm [8], [13], [14]. This difference in prevalence can mostly be attributed to the delineation of all four insertion sites of the mitral annulus in CMR while TTE studies primarily investigated the inferolateral site [8], [14]. When considering the prevalence of inferolateral MAD in the present study (59%, 7 mm extent, n = 54/91), results are similar to aforementioned TTE studies [8], [14] but higher than in Loeys-Dietz syndrome (LDS), a CTD due to mutations of the transforming growth factor beta signaling pathway, in which 37% of patients harbored inferolateral MAD (4 mm distance) in CMR [31]. The higher prevalence of MAD in MFS compared to LDS was also observed by Chivulescu et al., who reported a prevalence of MAD in 46% of patients with MFS and of 34% in LDS based on TTE [13]. Overall, prevalence of MAD in MFS in the present study was comparable to the UK Biobank CMR population study with 76% of participants harboring an MAD, albeit of smaller extent (3 mm) [7] and higher than in a study by Figliozzi et al., who found MAD in 49% of patients referred for clinically indicated CMR [32]. While MAD in MFS was most often observed at the inferior insertion (73%, n = 66/91), it was also common at other points along the hinge line of the PML (56%–62%), affecting the entire annulus in 40% (n = 36/91). In contrast, MAD in the UK Biobank cohort rarely affected at all sites (2%) and was most often found at inferior and anterior (58%–54%) insertions [7]. Rather than being interspersed with normal tissue, the high prevalence, large extent, and pan-annular distribution of MAD suggest a systemic pathology in MFS, potentially due to CTD-specific vulnerability and subsequent weakening of the mitral annulus.

No significant differences in left heart parameters and aortic diameters were observed between patients with and without MAD in the present study. These findings are in line with results in LDS [31], but contrary to observations in MFS based on TTE with Demolder et al. reporting LV dilatation, LV dysfunction, and increased aortic root z score in MAD [8]. While correlation between localizations and extent with aortic dilatation was mostly non-significant, MAD distance and the various insertion points yielded distinct, but significant correlations with changes to left heart parameters in the present study. Specifically, anterolateral and inferolateral insertion points as well as extent showed moderate correlation with reduced LVEF. While associations with LA dilatation were predominantly weak, insertion points and extent yielded moderate to strong correlations with LV dilatation. Besides underlining the benefits of the assessment of the separate insertion points and the extent of MAD in MFS using CMR [7], [12], [17], the results of this study indicate a potential interaction between MAD based on impairment of systolic annular contraction and mitral coaptation with LV dysfunction and dilatation over time [9].

Initially, MAD was described as a finding that is connected to MVP. However, MVP was found in 44.6% (n = 74/91) of MAD patients but was also observed in 11.8% (n = 2/17) of patients without MAD, underlining the fact that MAD and MVP have to be regarded as two separate disease entities. These findings are in line with observations in pediatric MFS patients (MVP in 48% of patients with MAD vs. 8% of patients without MAD [14]) with even higher occurrence in predominantly adult cohorts (71% of patients with MAD vs. 15% without MAD [8]). Reflecting the observed high occurrence of MVP in inferolateral MAD assessed by TTE [8], [14], involvement of inferolateral, anterior, and inferior insertion sites were strongly associated with MVP in the present study. These findings are partially in line with the UK Biobank cohort, in which inferolateral and inferior MAD but not anterior and anterolateral were associated with MVP [7]. These differences are mainly due to the overall impairment of the mitral annulus in MFS by MAD compared to the general population. Furthermore, the extent of MAD itself yielded a very strong correlation with MVP, in line with Dejgaard et al., who indicated a connection between MAD distance and patients’ age with development of MVP [12]. Since one could speculate that disjunctive areas of the annulus might represent points vulnerable to mechanical stress, especially in MFS with connective tissue weakness due to FBN1 mutations leading to annular instability, MAD may be regarded as a precursor for billowing, prolapse, and regurgitation. Interestingly, mitral valve billowing, reflecting abundant leaflet tissue without prolapse, exclusively occurred in patients with MAD, in line with the UK Biobank study [7] and not previously investigated in MFS before. Future longitudinal follow-up studies are required to investigate whether patients with MAD may develop concomitant billowing or prolapse with progression of disease severity in MFS over time.

There is a known association between inferolateral MAD and arrhythmic events, occurring in patients with [11] and without MVP [12]. In the present study, these were observed in 2% of all patients (n = 2/91) over a follow-up period of 4 years and were equally distributed between patients with and without MAD. These findings are partially in line with TTE-based studies: Chivulescu et al. found no significant difference (4% of the study population, follow-up of 5 years) between patients with and without MAD in MFS and LDS [13]. Conversely, these events (4%, follow-up of 12 years) exclusively occurred in MFS with MAD in the study by Demolder et al., yielding statistical significance [8]. MAD presence did not yield significant associations with aortic events in the present study (14% of the study population), in line with Demolder et al. (28%) [8] and Chivulescu et al. (67% in a surgically selected population) with the latter reporting a younger patient age at aortic event compared to those without MAD study [13]. Both Demolder et al. and Chivulescu et al. found that mitral events (5% [8] and 7% [13] in the overall cohorts) occurred significantly more often in MAD. The results of the present study corroborate these results with mitral valve surgery being only performed in patients with MAD, however not yielding statistical significance, mostly due to the limited number of events (2% of the overall population, n = 2/91). Of note, all-cause mortality did not differ between both groups in the present study, in line with Demolder et al [8].

Reflecting on the observed high prevalence of MAD based on the established threshold of ≥1 mm in the present study and previous works including healthy populations [7], [32], the identification of additional MAD features is necessary to detect the boundary between MAD as a normal finding and a disease. To this end, the performed subgroup analysis revealed that larger MAD extents were associated with significant changes to LV volumes and aortic dimensions. In this context, patients with MAD ≥8 mm yielded, besides known significantly higher prevalence of MVP in MAD patients, a larger displacement of MVP, increased LV volumes, and higher aortic root z scores. Furthermore, we identified a threshold of 7.1 mm MAD extent as a predictor for the presence of MVP. These findings can mostly be explained by general connective tissue weakness in MFS leading to dilative changes of the cardiovascular system and are in line with the study by Figliozzi et al., who also observed large MADs in patients with MVP [32]. Furthermore, we observed that patients treated with MVS during follow-up harbored a significantly larger MAD extent compared to MAD patients who did not receive surgery. However, given the very small number of MVS cases, these findings must be interpreted with caution. While we could not find significant differences in arrhythmic, aortic, or mitral events in the subgroup analysis, Figliozzi et al. indicated that patients harboring ≥ 6 mm MADs showed a trend towards a higher occurrence of arrhythmic events during a 12-month follow-up [32]. Overall, direct therapeutic implications of MAD extent remain unclear, requiring additional risk stratification [17] and further outcome studies.

This study carries several limitations. First, limitations due to the retrospective study design with an inherent risk of selection bias and missed data can be expected. In this context, 24-hour Holter monitoring was only performed in 14.3% of all patients (n = 13/91) in contrast to Demolder et al., where 73% of study participants received Holter monitoring. This difference is mainly attributed to the fact that Holter monitoring was only conducted in symptomatic individuals, while it was also performed annually within a research context in the study by Demolder et al. This has to be regarded as an important limitation of the present study, since arrhythmic events might have been missed. Second, the occurrence of outcomes was low, mostly due to the short follow-up period and above referenced limitation, questioning the robustness of the analyzed data and limiting subgroup analysis, especially regarding potential implications of inferolateral MAD, considered the clinically most important localization [17]. Third, we did not perform a comparison between CMR and TTE because examinations were not primarily focused on the assessment of MAD and since image acquisitions were not available for all patients due to the multicenter design. Fourth, neither LGE nor T1 mapping sequences were part of the CMR protocol at participating centers. Hence, we cannot provide data on the relationship between replacement fibrosis, mostly affecting the papillary muscles or adjacent inferolateral myocardium and associated with arrhythmic events in MVP [33] and MAD [12], with MAD morphology and outcome. Furthermore, an evaluation of potential links between interstitial fibrosis, associated with ventricular arrhythmias in MVP [34], and MAD was not feasible. Especially the assessment of both replacement and interstitial fibrosis preferably combined with longer-term outcome data may nurture future investigations and could aid in risk stratification of patients.

## 5 Conclusions

Based on multicenter CMR data, MAD was found to be highly prevalent in MFS, of large extent, mostly present at the inferior localization, and of pan-annular distribution, suggesting a systemic annular pathology. Overall presence of MAD was not associated with changes to left heart parameters or aortic dimensions. However, different localizations and extent of MAD yielded distinct correlations with LV dilatation and dysfunction and the presence of MVP, which was almost exclusively observed in MAD. Additionally, subgroup analysis revealed that patients with MAD ≥8 mm yielded a significantly larger displacement of MVP, increased LV volumes, and higher aortic root z scores. Cardiovascular events did not differ between patients with or without MAD regardless of applied thresholds for MAD extent. Nevertheless, MAD, especially of large extent, could serve as marker of disease progression in MFS, given shown associations with LV remodeling and dysfunction as well as aortic enlargement and the formation of MVP.

## Funding

This study received no specific funding.

## Author contributions

**Roman Johannes Gertz:** Writing – review & editing, Methodology, Formal analysis, Data curation. **Kenan Kaya:** Writing – original draft, Validation, Formal analysis, Data curation, Conceptualization. **Robert Terzis:** Writing – review & editing, Data curation. **Jonathan Kottlors:** Writing – review & editing, Writing – original draft, Methodology, Formal analysis, Data curation. **Carsten H. Gietzen:** Writing – original draft, Visualization, Validation, Project administration, Data curation, Conceptualization. **Lukas Goertz:** Writing – review & editing, Data curation. **Lenhard Pennig:** Writing – original draft, Visualization, Validation, Project administration, Formal analysis, Data curation, Conceptualization. **Jan Paul Janßen:** Writing – review & editing, Data curation. **Vanessa Schmidt:** Writing – review & editing, Data curation. **Thorsten W. Gietzen:** Writing – review & editing, Writing – original draft, Formal analysis, Data curation. **Leon Bischoff:** Writing – review & editing, Data curation. **Kilian Weiss:** Writing – review & editing, Data curation. **Christopher Hohmann:** Writing – review & editing, Data curation. **Jan M. Brendel:** Writing – review & editing, Data curation. **Patrick Krumm:** Writing – review & editing, Data curation. **Lukas Müller:** Writing – review & editing, Data curation. **Julian A. Luetkens:** Writing – review & editing, Supervision, Resources. **Philipp v Stein:** Writing – review & editing, Data curation. **David Maintz:** Writing – review & editing, Supervision, Resources. **Reza Dehdab:** Writing – review & editing, Data curation. **Tilman Emrich:** Writing – review & editing, Data curation. **Moritz C. Halfmann:** Writing – review & editing, Data curation.

## Ethics approval and consent

This retrospective study is part of the Radiological Cooperative Network project, which provides a collaborative environment for data exchange including all radiological departments of university hospitals in Germany. Given the retrospective study design and de-identification of patient data, this study was approved by the ethics committee of the leading center and the requirement for written informed consent was waived (24–1036-retro).

## Declaration of competing interests

The authors declare the following financial interests/personal relationships which may be considered as potential competing interests:Kenan Kaya reports administrative support and article publishing charges were provided by University Hospital Cologne Institute of Diagnostic and Interventional Radiology. Roman Johannes Gertz reports financial support was provided by Philips Healthcare Informatics Inc. Roman Johannes Gertz reports financial support was provided by Guerbet. David Maintz reports financial support was provided by Philips Healthcare Informatics Inc. Kilian Weiss reports financial support was provided by Philips Healthcare Informatics Inc. Tilman Emrich reports was provided by Siemens Healthineers AG. Tilman Emrich reports financial support was provided by Circle Cardiovascular Imaging Inc. Julian A. Luetkens reports financial support was provided by Bayer HealthCare AG. Julian A. Luetkens reports financial support was provided by GE Healthcare. Julian A. Luetkens reports financial support was provided by Novartis Pharmaceuticals Corporation. Julian A. Luetkens reports financial support was provided by Philips Healthcare Informatics Inc. Julian A. Luetkens reports financial support was provided by Siemens Healthineers AG. Lenhard Pennig reports financial support was provided by Philips Healthcare Informatics Inc. Lenhard Pennig reports financial support was provided by Guerbet. Kenan Kaya reports a relationship with University Hospital Cologne Institute of Diagnostic and Interventional Radiology that includes: employment. Kilian Weiss reports a relationship with Philips that includes: employment and equity or stocks. If there are other authors, they declare that they have no known competing financial interests or personal relationships that could have appeared to influence the work reported in this paper.

## Data Availability

The datasets generated and/or analyzed during the current study are not publicly available due to data protection, but are available from the corresponding author upon reasonable request.
