# Supplementary material for: Mitral annular disjunction in Marfan syndrome: a multicenter cardiovascular magnetic resonance study
Source: J Cardiovasc Magn Reson. 2025 Aug 13;28(1):101938. doi: 10.1016/j.jocmr.2025.101938 (PMC13265430; doi:10.1016/j.jocmr.2025.101938)
Supplement: Supplementary file 1 — Supplementary material [file mmc1.docx]

**Supplementary Material**

*Supplementary methods:*

|  | **1^st^ Center** | **2^nd^ Center** | **3^rd^ Center** | **4^th^ Center** |
| --- | --- | --- | --- | --- |
| Field of view [mm^2^] | 300-350 x 218-350 | 250-398 x 250-398 | 180-350 x 200-350 | 273-356 x 340-399 |
| Resolution |  |  |  |  |
| Acquired [mm^2^] | 1.7-1.8 x 1.7-1.9 | 1.8-2.0 x 1.7-2.9 | 1.7-1.8 x 1.7-2.3 | 1.7-2.0 x 1.7-2.3 |
| Reconstructed [mm^2^] | 1.0 x 1.0 | 0.9-1.5 x 0.9-1.5 | 0.7-1.5 x 0.7-1.5 | 1.5 x 1.5 |
| Slice thickness [mm] | 8.0 | 6.0-8.0 | 6.0-8.0 | 8.0 |
| Repetition time [ms] | 3.0 | 2.5-4.0 | 2.5-4.0 | 4.0 |
| Echo time [ms] | 1.5 | 1.3-2.0 | 1.2-2.0 | 1.4-1.6 |
| Flip angle [°] | 60 | 40-60 | 40-70 | 38-51 |
| Temporal resolution [ms]* | 25 | 25-40 | 25-40 | 38-44 |
| Acceleration | Compressed SENSE,  factors 4-4.4 | SENSE,  factors 1-5.7 | GRAPPA and compressed sensing,  factors 2-6 | Compressed sensing,  factor 6.5 |

Supplementary Table 1: Balanced steady-state free precession cine parameters as acquired at the four participating centers. *=heart rate dependent (provided value refers to a heart rate of 80 beats per minute). GRAPPA=GeneRalized Autocalibrating Partial Parallel Acquisition. SENSE=sensitivity encoding. 1^st^ Center: Institute for Diagnostic and Interventional Radiology, Faculty of Medicine and University Hospital Cologne, University of Cologne, Cologne, Germany. 2^nd^ Center: Department of Diagnostic and Interventional Radiology, University Hospital Bonn, Bonn, Germany. 3^rd^ Center: Department of Radiology, Diagnostic and Interventional Radiology, University of Tübingen, Tübingen, Germany. 4^th^ Center: Department of Diagnostic and Interventional Radiology, University Medical Center of the Johannes Gutenberg-University, Mainz, Germany.

*Supplementary results:*

| **Scanner model** | Patients, n (%) |
| --- | --- |
| Philips Ingenia | 72 (79.1) |
| Philips Ingenia Elition X | 1 (1.1) |
| Philips Intera | 8 (8.8) |
| Siemens Espree | 1 (1.1) |
| Siemens Prisma | 8 (8.8) |
| Siemens Sonata | 1 (1.1) |
| **Field strength** |  |
| 1.5 T | 90 (98.9) |
| 3.0 T | 1 (1.1) |
| **MRA sequence** |  |
| REACT | 29 (31.9) |
| 3D CE-MRA | 34 (37.4) |
| 4D CE-MRA | 20 (22.0) |
| Steady-state 3D CE-MRA | 8 (8.8) |
| **Participating centers** |  |
| 1^st^ Center | 59 (64.8) |
| 2^nd^ Center | 21 (23.1) |
| 3^rd^ Center | 6 (6.6) |
| 4^th^ Center | 5 (5.5) |

Supplementary Table 2: Employed CMR scanner models (manufactured by Philips Healthcare, Best, The Netherlands and Siemens Healthineers, Erlangen, Germany), field strength, MRA sequences, and number of examinations performed at respective centers. CE-MRA=contrast-enhanced magnetic resonance angiography. REACT=Relaxation-Enhanced Angiography without Contrast and Triggering. 1^st^ Center: Institute for Diagnostic and Interventional Radiology, Faculty of Medicine and University Hospital Cologne, University of Cologne, Cologne, Germany. 2^nd^ Center: Department of Diagnostic and Interventional Radiology, University Hospital Bonn, Bonn, Germany. 3^rd^ Center: Department of Radiology, Diagnostic and Interventional Radiology, University of Tübingen, Tübingen, Germany. 4^th^ Center: Department of Diagnostic and Interventional Radiology, University Medical Center of the Johannes Gutenberg-University, Mainz, Germany.
